# Stereoisomer-dependent conversion of dinaphthothienothiophene precursor films

**DOI:** 10.1038/s41598-022-08505-5

**Published:** 2022-03-15

**Authors:** Nobutaka Shioya, Masamichi Fujii, Takafumi Shimoaka, Kazuo Eda, Takeshi Hasegawa

**Affiliations:** 1 Institute for Chemical Research, Kyoto University, Gokasho, Uji, Kyoto 611-0011 Japan; 2 Department of Chemistry, Graduate School of Science, Kobe University, 1-1 Rokko-dai, Nada-ku, Kobe, Hyogo 657-8501 Japan

**Keywords:** Chemistry, Materials science

## Abstract

Soluble precursor materials of organic semiconductors are employed for fabricating solution-processable thin film devices. While the so-called precursor approach has already been tried for various organic electronic devices such as transistors and solar cells, understanding of the conversion process in the film lags far behind. Here, we report that molecular aggregation of the precursor compound significantly influences the thermal conversion reaction in the film. For this study, two stereoisomers of a dinaphthothienothiophene (DNTT) precursor that are the endo- and exo-DNTT-phenylmaleimide monoadducts are focused on. The structural change during the thermal conversion process has been investigated by a combination of infrared spectroscopy and X-ray diffraction techniques. The results show that the endo-isomer is readily converted to DNTT in the film by heating, whereas the exo-isomer exhibits no reaction at all. This reaction suppression is found to be due to the self-aggregation property of the exo-isomer accompanying the intermolecular C–H$\cdots$O interactions. This finding shows a new direction of controlling the on-surface reaction, as well as the importance of analyzing the film structure at the initial stage of the reaction.

## Introduction

On-surface synthesis has emerged as a promising approach for preparing two-dimensional nanosheet structures such as covalent organic frameworks and graphene nanoribbons^1–4^. In addition, this solid-state reaction-based technique is important for synthesis of compounds that cannot be produced in solution, as found for linear acenes fused with seven or more benzene rings^5^. This approach is also useful for fabricating solution-processed thin-film electronic devices^6–13^. In this case, the desired material is obtained in the film by heating or photo-irradiating a solvent-soluble precursor compound. The precursor approach is the only method by which films of insoluble compounds can be obtained by a wet process.

From the viewpoint of crystal growth, the precursor approach is substantially different from conventional techniques such as physical vapor deposition. In the case of a vapor-deposited pentacene film, immediately after deposition on the substrate, a polycrystalline film is formed with the molecular axis perpendicular to the film surface^14–17^. The pentacene molecules that can thermally be converted from a precursor compound, on the other hand, exhibit stepwise crystal growth beginning with a random orientation^18,19^. At the early stage of the reaction, the residual unreacted molecules make the self-aggregation of the reaction product poor to produce a randomly oriented film. When the thermal energy on heating is provided sufficiently to complete the chemical conversion, a highly ordered polycrystalline film is formed. Therefore, control of the on-surface reaction is essential for obtaining the desired film structure.

**Figure 1:**
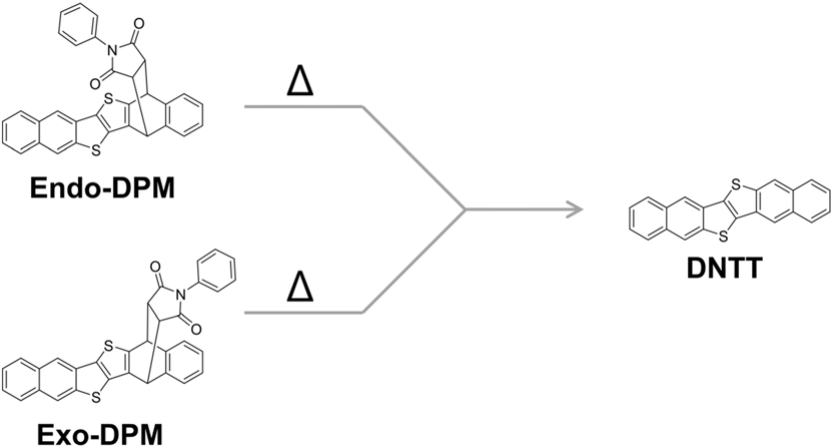
Structural conversion reaction of DPM to DNTT.

Considering that the chemical conversion proceeds as a solid-phase reaction, the molecular aggregation of the precursor compound before the thermal treatment should influence the efficiency of the chemical reaction. In other words, the conversion reaction with the precursor approach would be controlled by changing the aggregation structure of the precursor compound in the film. To demonstrate this expectation, we focus on the crystalline precursor materials^9,10^ of dinaphtho[2,3-b:2’,3’-f]thieno[3,2-b]thiophene (DNTT; Fig. 1), which is a high-performance semiconducting material with excellent carrier mobility^20–23^. The precursor compound, DNTT-phenylmaleimide monoadduct (DPM), has several stereoisomers, of which the endo (5,14-) and exo (5,14-) isomers (hereafter denoted as endo-DPM and exo-DPM, respectively; Fig. 1) are typically used for organic field-effect transistors (OFET)^9,10,24,25^. According to the original papers^9,10^, exo-DPM has a lower solubility in common organic solvents than endo-DPM. This suggests that the exo-isomer takes a tighter packing in a solid sample than the endo-isomer, and the difference in the aggregation structure would have a significant impact on the conversion reaction to DNTT. The difference of the on-surface conversion reaction, however, has not been studied so far.

In the present study, the thermal conversion processes of the two stereoisomers in thin films are investigated by means of ex-situ specular X-ray diffraction (XRD), grazing incidence XRD (GIXD), and p-polarized multiple-angle incidence resolution spectrometry (pMAIRS)^26,27^. The combination analysis has revealed that the exo-isomer takes a close packing in the film as expected, so that it does not show the thermal conversion reaction to DNTT.

## Results and discussion

**Figure 2:**
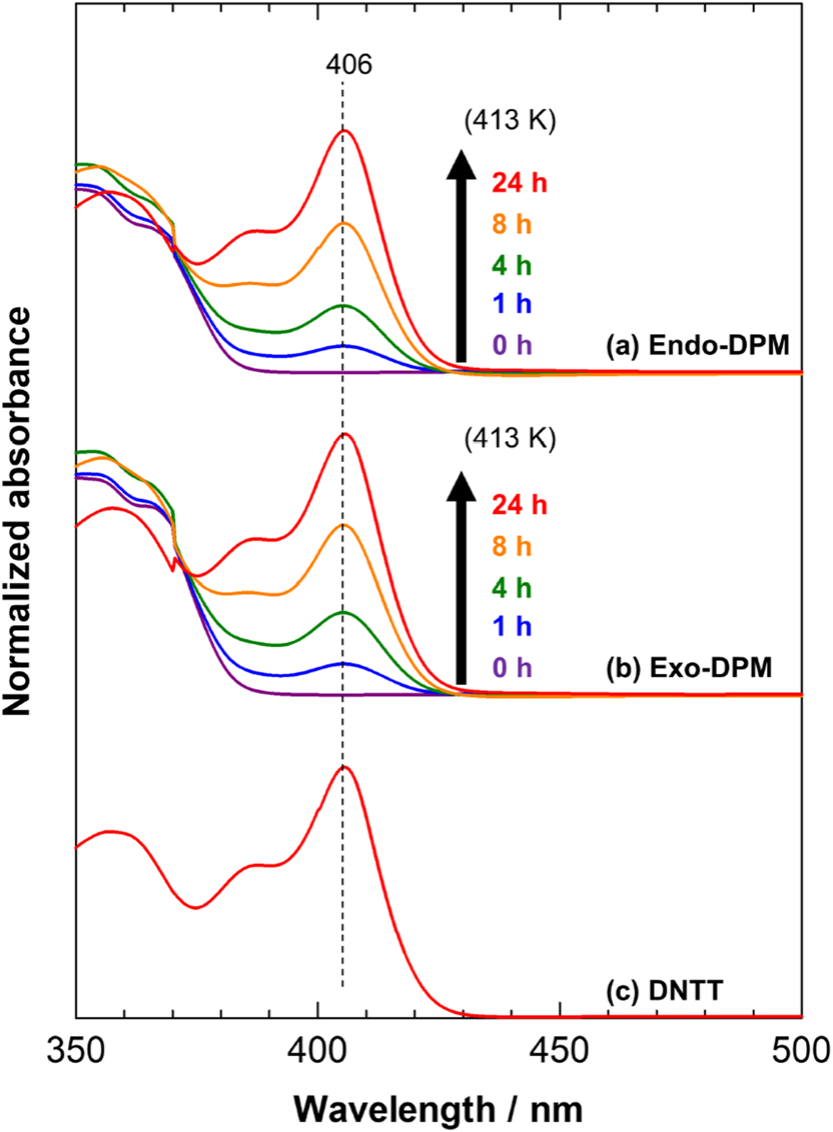
Annealing-time-dependent UV–Vis spectra of precursor solutions (**a**,**b**) and the reference spectrum of a DNTT solution (**c**).

Before discussing the molecular aggregation structure in thin films, we confirm the thermal conversion reaction from DPM to DNTT. Figure 2 presents the ultraviolet–visible (UV–Vis) spectra of solutions of the endo- and exo-isomers at several annealing times. The time-dependent spectra are very common to the two isomers: both isomers show a continuous increase in the peak intensity at 406 nm with time (Fig. 2a,b). By comparing these spectra with that of a pure DNTT solution (Fig. 2c), the band can be readily assigned to DNTT. After thermal annealing at 413 K for 24 h, the spectra of the precursor solutions are almost identical in shape to the reference spectrum (Fig. 2), indicating that the conversion reaction proceeds quantitatively in “solution” for both isomers.

### Conversion reaction of the endo-isomer

**Figure 3:**
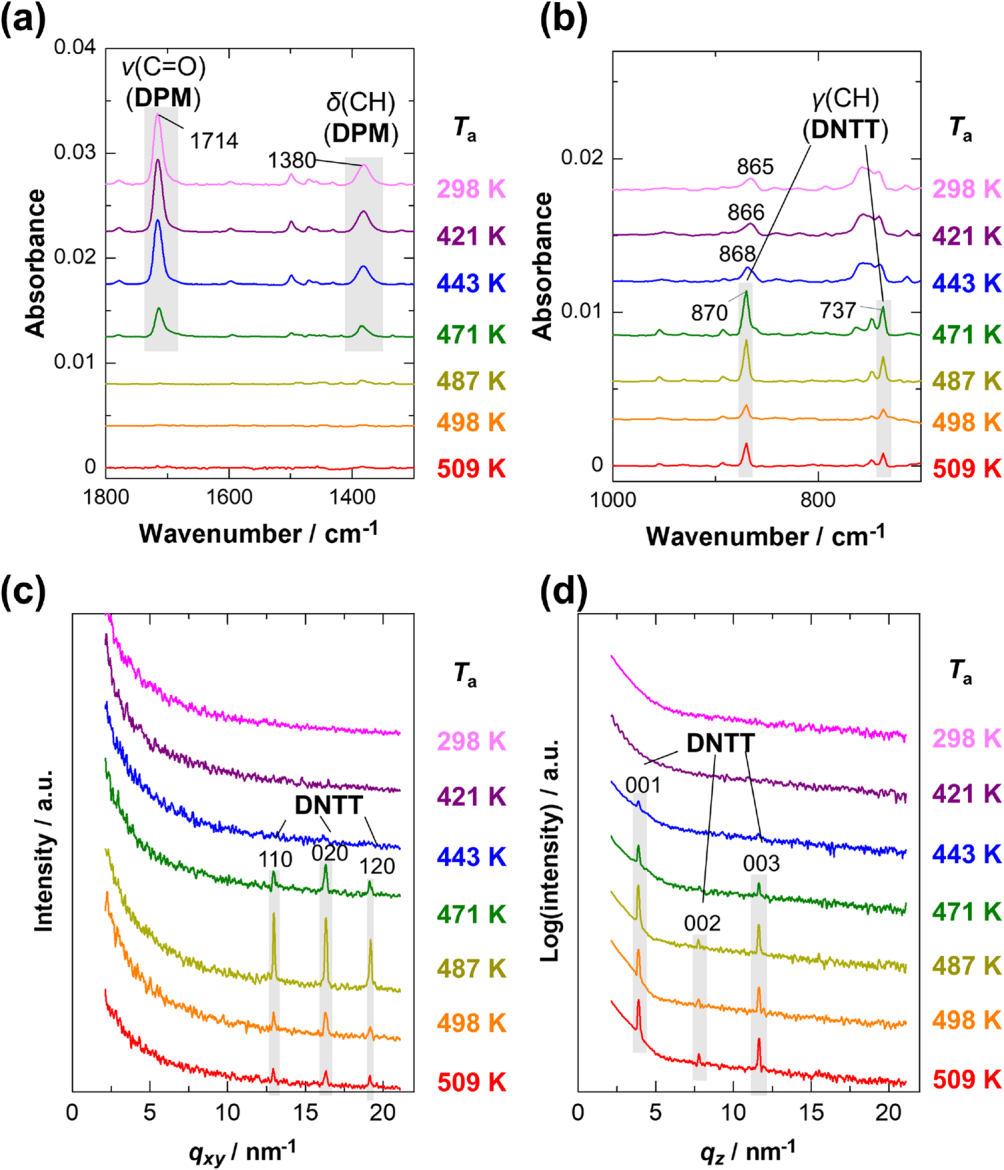
Orientation-free IR spectra (**a**,**b**), GIXD patterns (**c**), and specular XRD patterns (**d**) of endo-DPM thin films annealed for 10 min as a function of annealing temperature (*T*_a_).

We first discuss the thermal conversion reaction of the endo-isomer in a thin film. Figure 3a,b shows infrared (IR) spectra of the films as a function of annealing temperature (*T*_a_), in which the molecular orientation is annihilated and only the quantity of chemical species can be quantitatively discussed. These “orientation-free spectra” are readily calculated by averaging the pMAIRS-IP (in-plane) and pMAIRS-OP (out-of-plane) spectra (Fig. S1a) in accordance with our previous work^19^. Judging from the simulated spectrum by the density functional theory (DFT) calculations (Fig. S2), the strong bands at 1714 cm^−1^ and 1380 cm^−1^ in Fig. 3a are assigned to the C=O stretching vibration (ν(C=O)) and C–H in-plane deformation vibration (δ(CH)) modes, respectively, of the leaving group. In the lower wavenumber region (Fig. 3b), on the other hand, the C–H out-of-plane deformation vibration (γ(CH)) bands of the reaction product (DNTT) are observed at 870 cm^−1^ and 737 cm^−1^ (for the assignments see Fig. S2). In these spectra, the bands of the precursor clearly decrease with increasing *T*_a_ and disappear at 487 K; instead, the γ(CH) bands of DNTT develop, which straightforwardly confirms the chemical conversion from endo-DPM to DNTT. Note that the intensity of the γ(CH) bands of DNTT reaches its maximum at *T*_a_ = 471 K, where the conversion is not completed yet, and turns into a decreasing trend above this temperature. This indicates that heating at a high temperature induces sublimation of the product, DNTT, which supports previously reported thermogravimetric results^9,10^.

The result is further confirmed by the GIXD and specular XRD patterns in Fig. 3c,d, respectively. For the as-spun film (*T*_a_ = 298 K), both diffraction patterns show no signals, indicating that the precursor compound is in an amorphous state. When the film is annealed at 443 K or higher, several diffraction peaks appear in the patterns. According to literature^23,28^, these peaks are all attributed to the Bragg reflections of DNTT as indexed in the figure. In this manner, the “amorphous” endo-isomer is transformed into the target compound by heating.

As found in Fig. 3d, the 00* l* peak series are selectively observed by XRD under specular-reflection conditions, in which diffractions along the surface perpendicular direction appear specificaly. The molecules in the crystallite are thus found to have a standing-up orientation in the films, since the c*-axis is almost parallel to the molecular axis^23^. In addition, based on the position of the 003 peak, the interlayer spacing is calculated to be 1.620 nm, which is consistent with the previously reported values of the bulk structures (1.621 or 1.624 nm)^23,28^ rather than the thin-film phase (1.633 nm)^28^. This result is acceptable given that this reaction occurs everywhere in the film, not limited near the substrate surface.

### Suppression of conversion reaction of the exo-isomer

**Figure 4:**
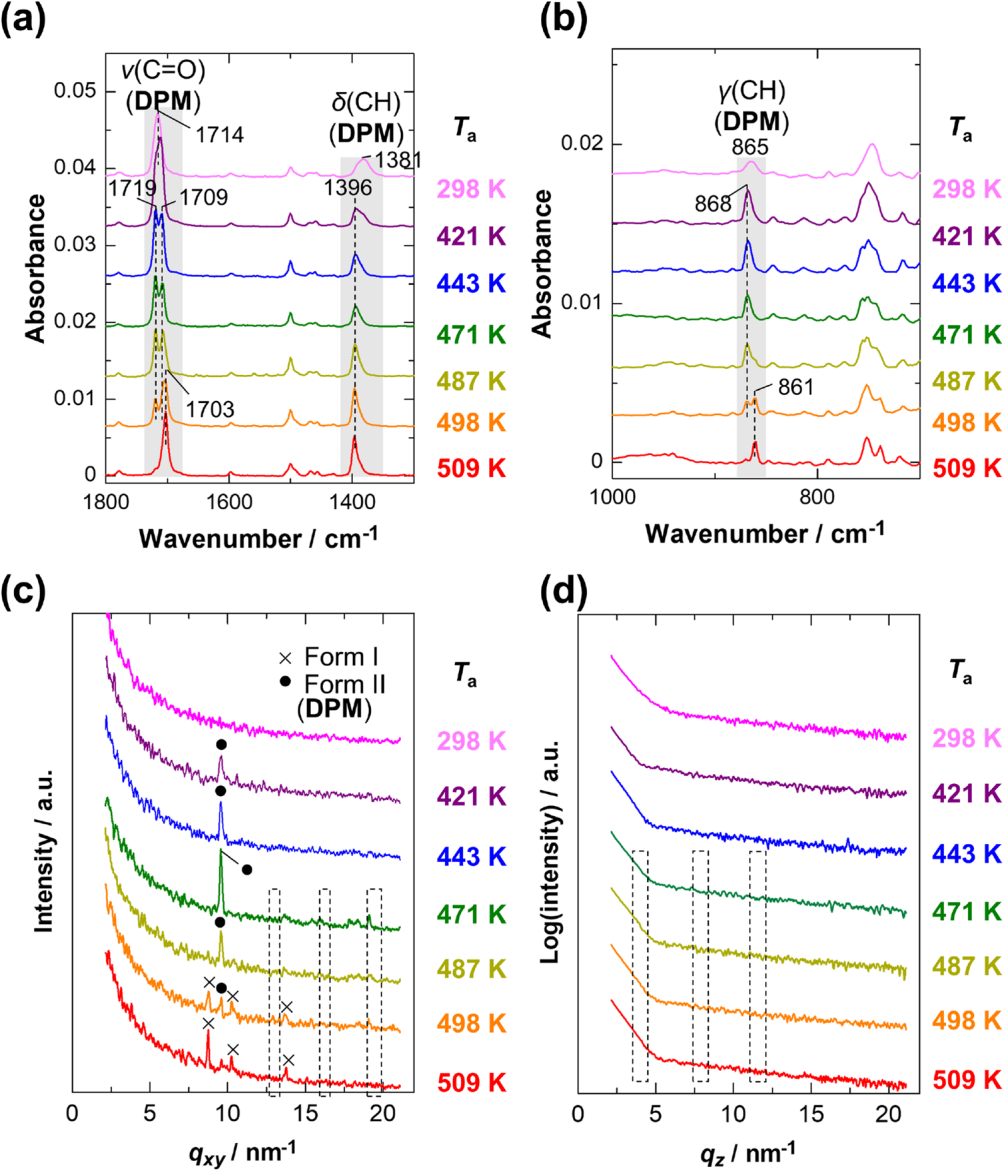
Orientation-free IR spectra (**a**,**b**), GIXD patterns (**c**), and specular XRD patterns (**d**) of exo-DPM thin films annealed for 10 min as a function of annealing temperature (*T*_a_). The areas enclosed by dashed lines in the diffraction patterns indicate the peak positions of DNTT.

The structure of the exo-isomer film is also analyzed to study the influence of stereoisomerism of DPM on the thermal conversion reaction. The IR spectra and the XRD patterns of the as-spun film in Fig. 4 are similar to those of the endo-isomer film. Such kinetically favorable amorphous films are typically obtained by spin-coating using highly volatile solvents such as chloroform^29,30^. As a consequence, the difference in molecular aggregation between the two stereoisomers is not found in the as-spun films. The annealed films, on the other hand, yield totally different results from the endo-isomer films. To our surprise, no peaks of DNTT are found in either the IR or XRD data of the exo-isomer samples (Fig. 4), although the chemical conversion is believed to proceed by heating at $\gtrsim$ 473 K^10^. The exo-isomer, in this manner, does not react thermally at all in films, which is largely different from the endo-isomer. Note that OFET devices fabricated from exo-DPM are reported to have lower carrier mobility than those fabricated from endo-DPM^9,10^. The present results readily explain the poor device performance of OFETs using exo-DPM.

The GIXD patterns show a single sharp peak in the temperature range of 421 K ≤ *T*_a_ ≤ 487 K, while different peaks are observed at the elevated temperature of 509 K in Fig. 4c. At the intermediate temperature (*T*_a_ = 498 K), the diffraction pattern is composed of the two patterns. This indicates that exo-DPM changes its crystalline polymorphs depending on *T*_a_, but the expected thermal conversion does not occur. Since the peak locations of the film annealed at 509 K coincide with those of a powder sample (Fig. S3), the precursor is suggested to change from a metastable state to a thermodynamically stable state having the same crystal structure as the known bulk structure^10^ (denoted as “form I”). The diffraction peak of the metastable one, on the other hand, is a newly found one in this study, and we call this crystal structure "form II."

Focusing on the ν(C=O) band around 1710 cm^−1^ in the IR spectra (Fig. 4a), the band splits into two components at 1719 cm^−1^ and 1709 cm^−1^ in the temperature range of 443 K ≤ *T*_a_ ≤ 498 K. By comparison with the corresponding diffraction patterns, this spectral change is attributed to crystallization without thermal conversion. As shown in Fig. S1b, the higher-wavenumber component appears predominantly in the pMAIRS-OP spectra, whereas the other component is observed mainly in the pMAIRS-IP spectra. This suggests that directions of the transition moments of these vibrational modes are largely different from each other, which may be explained by the Davydov splitting due to the intermolecular vibrational coupling^31^. Therefore, the crystal structure of form II is uniquely found in a thin film in the temperature range, which would be characterized by the presence of two (or more) translationally inequivalent molecules in the unit cell, in contrast to the known bulk structure (form I)^10^. At the elevated temperature of 509 K, as expected, the ν(C=O) band gets back to a single peak (Fig. 4a), reflecting the structural conversion into the form I polymorph. In a similar manner, the polymorphs can also be identified by using the γ(CH) band of DPM; the band locations of 868 cm^−1^ and 861 cm^−1^ in Fig. 4b correspond to the form II and form I structures, respectively. Another noteworthy feature is the shift of the δ(CH) band upon crystallization (Fig. 4a), which will be discussed later.

These experimental observations additionally show that in the films annealed at *T*_a_ = 421 K, the exo-isomer molecules have already been crystallized (Fig. 4), while the endo-isomer ones retain an amorphous structure (Fig. 3). This result implies that the structural conversion to DNTT is strongly restricted in highly ordered aggregates. In other words, the self-aggregation property suppresses the conversion reaction. The same conclusion can also be drawn from the data measured as a function of annealing time (Figs. S4–S6).

### Impact of stereoisomer on the molecular aggregation structure

**Figure 5:**
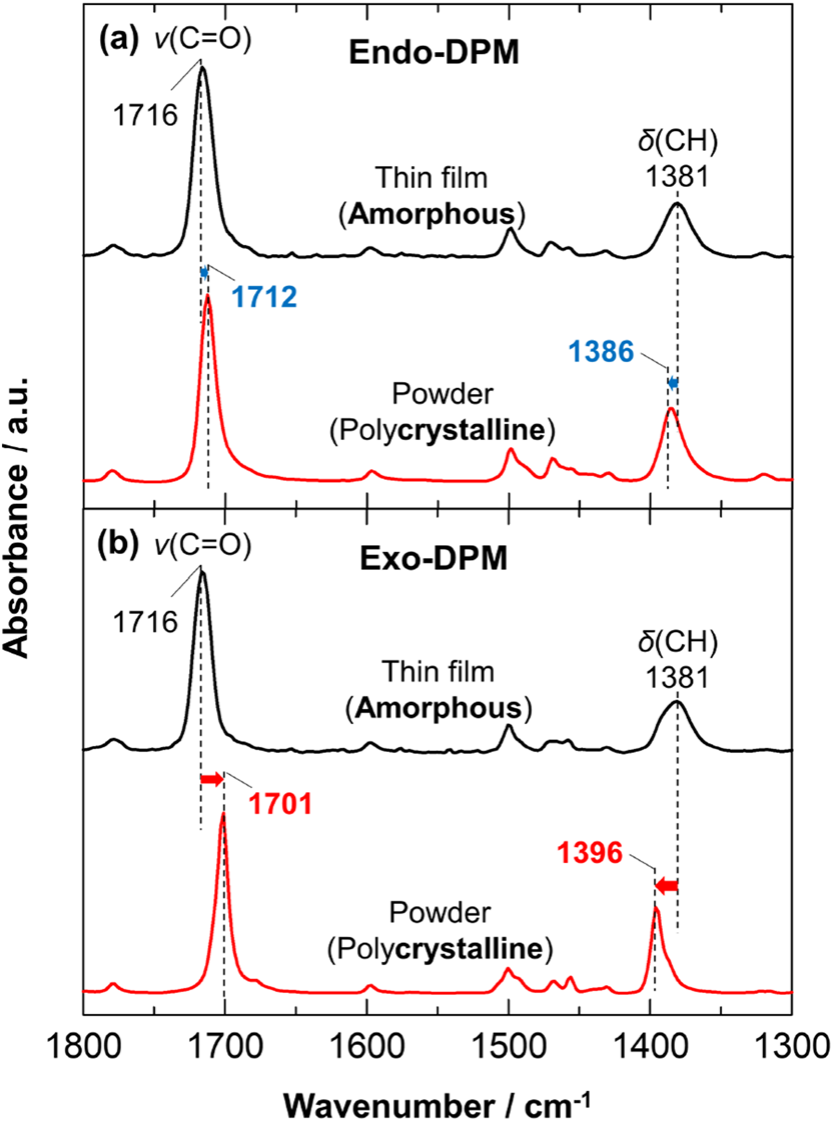
IR ATR spectra of polycrystalline powder samples of endo-DPM (**a**) and exo-DPM (**b**), and the orientation-free spectra of the amorphous films in Figs. 2 and 3.

**Figure 6:**
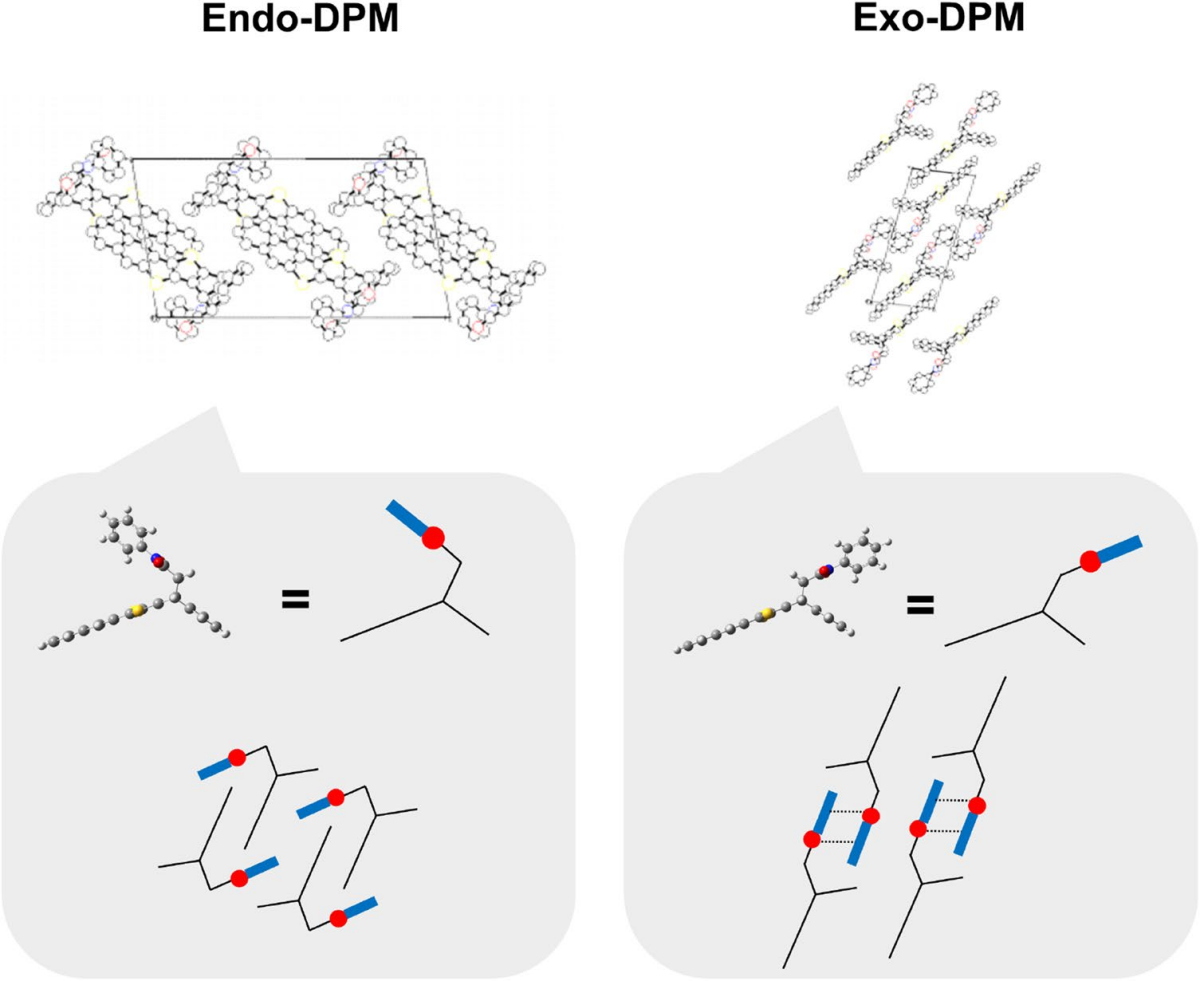
Schematics of the known crystal structures of endo-DPM and exo-DPM. Top schematic reprinted with permission from the following article: Kimura et al.^10^, Figs. S5 and S7 (2015). ( Copyright 2014 WILEY–VCH Verlag GmbH & Co. KGaA.)

To clarify the reason why the exo-isomer exhibits a higher aggregation property than the endo-isomer, the known crystal structures of the two isomers are compared based on their IR spectra. Figure 5 shows the IR spectra of polycrystalline powder samples of these isomers as well as the amorphous films. The band positions of the bulk samples depend greatly on the stereoisomers, as typically found for the ν(C=O) and δ(CH) bands. This should reflect the difference in the molecular packing, i.e., the crystal structure, since the amorphous films show no difference in the spectra. Of note is that for both isomers, the ν(C=O) band of the bulk sample is shifted to a “lower” wavenumber position than that of the amorphous film, whereas the δ(CH) band exhibits a shift to a “higher” position particularly found in Fig. 5b. Bantignies and co-workers reported that the ν(C=O) and δ(NH) (N–H in-plane deformation vibration) bands of urea-based hybrid silica shift to lower and higher positions, respectively, due to formation of hydrogen bonds between the urea groups^32^. Based on this observation, the band shifts found in Fig. 5 strongly suggest intermolecular hydrogen bonding between the carbonyl and phenyl C–H groups. The C–H$\cdots$O interactions of aromatic C–H donors have been recognized by several experimental and computational studies^33–38^. Here, the shifts of both vibrational bands of the exo-isomer are larger than those of the endo-isomer. This implies that the hydrogen bond of the exo-isomer is relatively strong. This experimental fact is understandable by considering the crystal structures depicted in Fig. 6, in which the leaving groups of the exo-isomers are fairly close to each other. Based on this observation, the high-wavenumber shift of the δ(CH) band found in the spectra of the exo-isomer films would also indicate the formation of hydrogen bonds (Fig. 4a). The intermolecular hydrogen bonding between the leaving groups is thus concluded to be a reason for the high aggregation property of the exo-isomer.

### Mechanism of thermal conversion reaction

Finally, we discuss the reaction mechanism of the structural conversion from DPM to DNTT with regard to the retro-Diels–Alder reaction. This reaction is known to be irreversible, but details of the reaction mechanism have not yet been elucidated. A previous study reported that the reaction of a diketone precursor involves the cleavage of the C–C bond between one of the bridgeheads and the neighboring carbonyl group to form a biradical intermediate^11^. Since the biradical one is highly reactive, the reverse reaction can occur as long as the leaving group remains in the same position. Molecular dynamics simulations show that the retro-Diels–Alder reaction takes several hundred femtoseconds^11^. If the leaving group can move beyond a distance greater than the van der Waals radius during this time, the reaction is expected to proceed in the forward direction. Once the precursor compound is crystallized, however, the leaving group would stay unmoved, and the conversion reaction would be suppressed accompanying the rapid reverse reaction from the intermediate state.

## Conclusions

We have revealed the film growth processes of the DNTT precursor, DPM, depending on the stereoisomerism. Now, the exo-isomer should be emphasized not convertible to DNTT “in films,” although DPM is commercially announced as a useful precursor material for obtaining DNTT by thermal treatment on surface, which has proven to be incorrect in this study. Another notable result is the identification of the C–H$\cdots$O interaction between the leaving groups using IR spectroscopy. Throughout this study, the stereoisomerism of the precursor compound is found to have a significantly large impact on crystallization and on the conversion reaction to DNTT. This study is believed to be an important step for controlling various on-surface reactions.

## Methods

### Film preparation

Powder samples of endo-DPM (purity: > 97.0%, catalogue no. D5153) and exo-DPM (purity: > 95.0%, catalogue no. D5154) purchased from Tokyo Chemical Industry (Tokyo, Japan) were used without additional purification. The samples were dissolved in chloroform at a concentration of 0.2 wt%. Since exo-DPM was not completely dissolved at room temperature, it was dissolved by heating and stirring the solution at about 80 °C; then the resulting solution was got back to room temperature to prepare spin-coated films. The films were formed by dropping 20 µL of the solution onto a silicon (Si) substrate at a spinning speed of 1000 rpm using an Active (Saitama, Japan) ACT-300T spin coater. For the substrate, a double-side polished Si wafer with a thickness of 625 ± 25 µm was used with surface-oxidization treatments. The spin-coated films were subjected to thermal annealing at different temperatures for 10 min each. The temperature was measured directly on the substrate surface using a thermocouple. In addition, to investigate the effect of treatment time (*t*), thin films were also prepared by annealing at the fixed temperature of 471 K for *t* = 1, 10, and 30 min. All the films were heated by putting them directly on a preheated stage. The obtained films were subjected to XRD and pMAIRS measurements under ambient conditions.

### XRD measurements

XRD measurements of the sample films were performed on a Rigaku (Tokyo, Japan) SuperLab^39^ X-ray diffractometer equipped with a parabolic multilayer X-ray mirror and a scintillation detector. The characteristic X-rays were Cu Kα rays (0.15418 nm) generated by a rotating anode X-ray generator, where the tube voltage and current were set to 40 kV and 30 mA, respectively. For the specular XRD measurement, the detector and sample stage were scanned symmetrically and simultaneously, so that the scattering angle was always twice the incident angle. For the GIXD in-plane measurement, the incident angle was fixed at 0.20°, and only the detector was scanned in the film plane. The obtained diffraction patterns were smoothed by the Savitzky-Golay method involved in the Rigaku PDXL software. The number of points for smoothing was set to 11.

### IR pMAIRS measurements

For the pMAIRS measurements, a Thermo Fisher Scientific (Madison, WI, USA) Magna 550 FT-IR spectrometer equipped with an automatic MAIRS accessory (TN 10-1500) was used. To make the p-polarized light, a PIKE Technologies (Madison, WI, USA) wire-grid polarizer built on germanium (Ge; 090-1500) was equipped in the sample chamber. The diameter of the aperture and the wavenumber resolution were set to 150% and 4 cm^−1^, respectively. The transmitted IR-ray was detected by a mercury-cadmium-telluride (MCT) detector. The angle of incidence was set to 9 through 44° by 5° intervals, and the accumulation number of the interferogram was set to 500.

### IR ATR measurements

IR attenuated total reflection (ATR) measurements were performed using a Thermo Fischer Scientific Nicolet 6700 FT-IR spectrometer equipped with a Spectra-Tech (Oak Ridge, TN, USA) Foundation Thunder Dome ATR accessory. The accessory was a single reflection type with an angle of incidence of 45°, and the prism was made of Ge. The unpolarized IR-ray was detected by a MCT detector. The aperture diameter and the wavenumber resolution were set to 34% and 1 cm^−1^, respectively. The number of scans was set to 256. The band assignments were made on the DFT calculations, which were performed with a basis set of B3LYP/6-31G(d) using the Gaussian 09 software^40^. The calculated wavenumber, ${\nu }_{\text{calc}}$, was corrected using the previously reported empirical equation^41^:

1 $${\nu }_{\mathrm{calc}}^{^{\prime}}={\nu }_{\mathrm{calc}}(1.0-0.00001692{\nu }_{\mathrm{calc}})$$

where ${\nu }_{\text{calc}}^{^{\prime}}$ is the corrected wavenumber.

### UV–Vis measurements

The UV–Vis spectra of solution samples were measured using a JASCO (Tokyo, Japan) V-630 UV–Vis double-beam spectrometer. A solution cell with an optical path length of 1 cm was used, and the wavelength range was set to 290–800 nm with a scanning speed of 200 nm min^−1^. Powder samples of endo-DPM and exo-DPM were dissolved in 1,2,4-trichlorobenzene at a concentration of 1.0 × 10^–3^ wt% for the measurements. Each solution was heated at 431 K in an oil bath, where the temperature in the bath was measured using a mercury thermometer. Several solution samples with different treatment periods of time (1, 4, 8, and 24 h) were prepared, and they were all measured.

## Supplementary Information

Supplementary Information.
