# Supplementary material for: Stereoisomer-dependent conversion of dinaphthothienothiophene precursor films
Source: Sci Rep. 2022 Mar 15;12:4448. doi: 10.1038/s41598-022-08505-5 (PMC8924201; doi:10.1038/s41598-022-08505-5)
Supplement: Supplementary file 1 — Supplementary Information. [file 41598_2022_8505_MOESM1_ESM.pdf]

Supplementary Information

Stereoisomer-Dependent Conversion of Dinaphthothienothiophene Precursor Films

Nobutaka Shioya[1*], Masamichi Fujii[1], Takafumi Shimoaka[1], Kazuo Eda[2], and Takeshi Hasegawa[1*]

[1]Institute for Chemical Research, Kyoto University, Gokasho, Uji, Kyoto 611-0011, Japan

[2]Department of Chemistry, Graduate School of Science, Kobe University, 1-1 Rokko-dai, Nada-ku, Kobe, Hyogo 657-8501, Japan

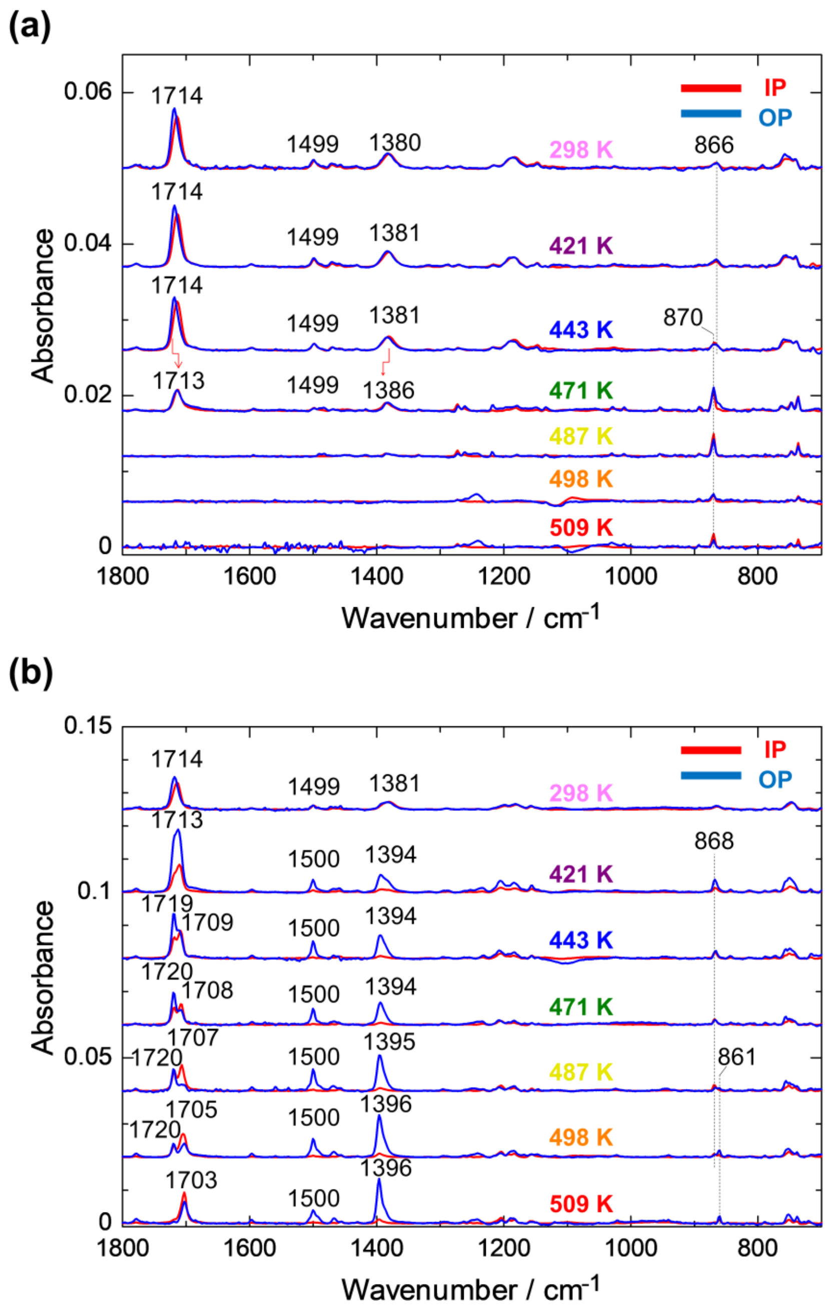

Figure S1. IR pMAIRS spectra of endo-DPM (a) and exo-DPM (b) thin films annealed for 10 min as a function of annealing temperature. The red and blue lines correspond to the in-plane (IP) and out-of-plane (OP) spectra, respectively.

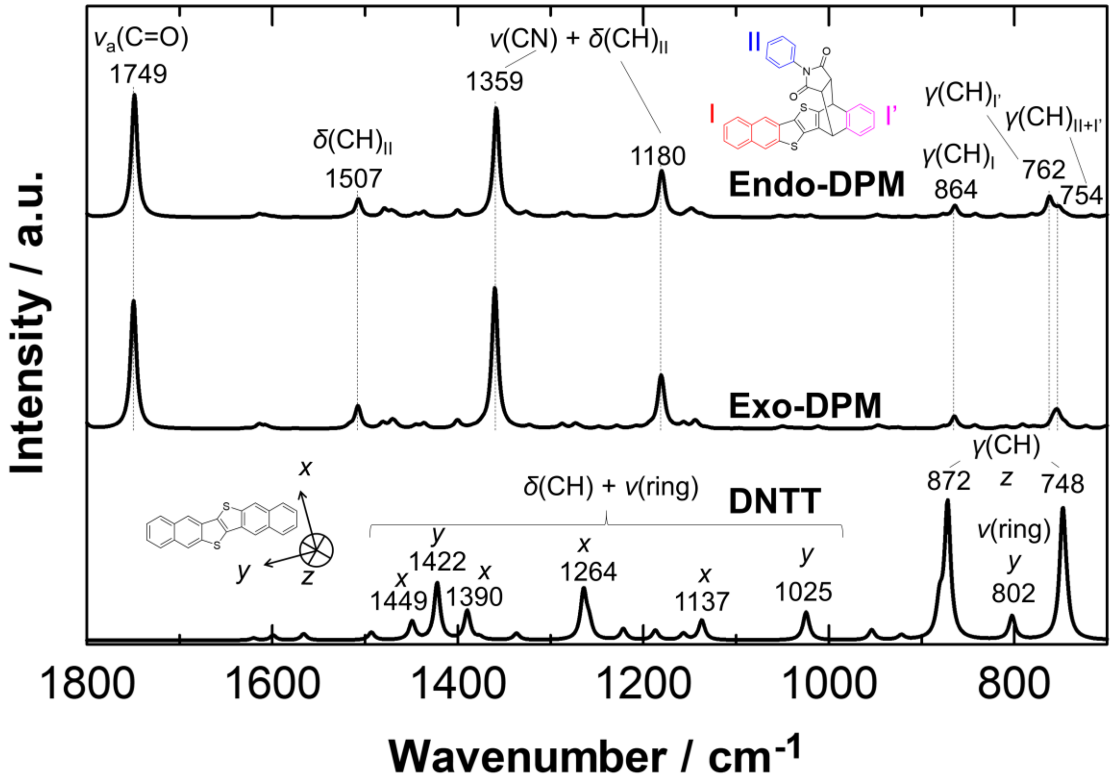

Figure S2. Simulated spectra of endo-DPM, exo-DPM, and DNTT predicted by the DFT calculation with a basis set of B3LYP/6-31G(d).

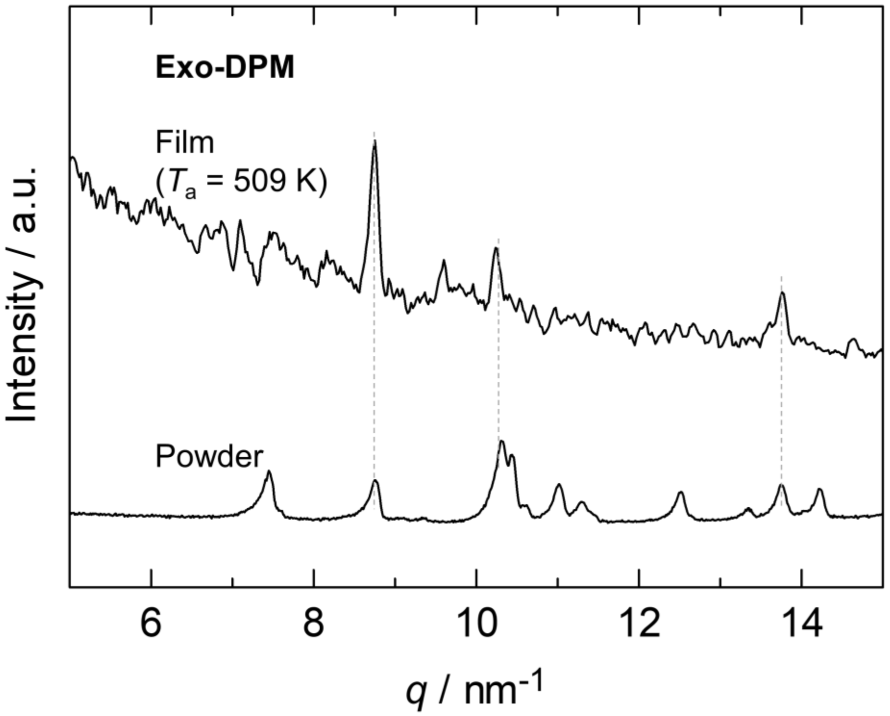

Figure S3. XRD patterns of a powder sample and a thin film of exo-DPM.

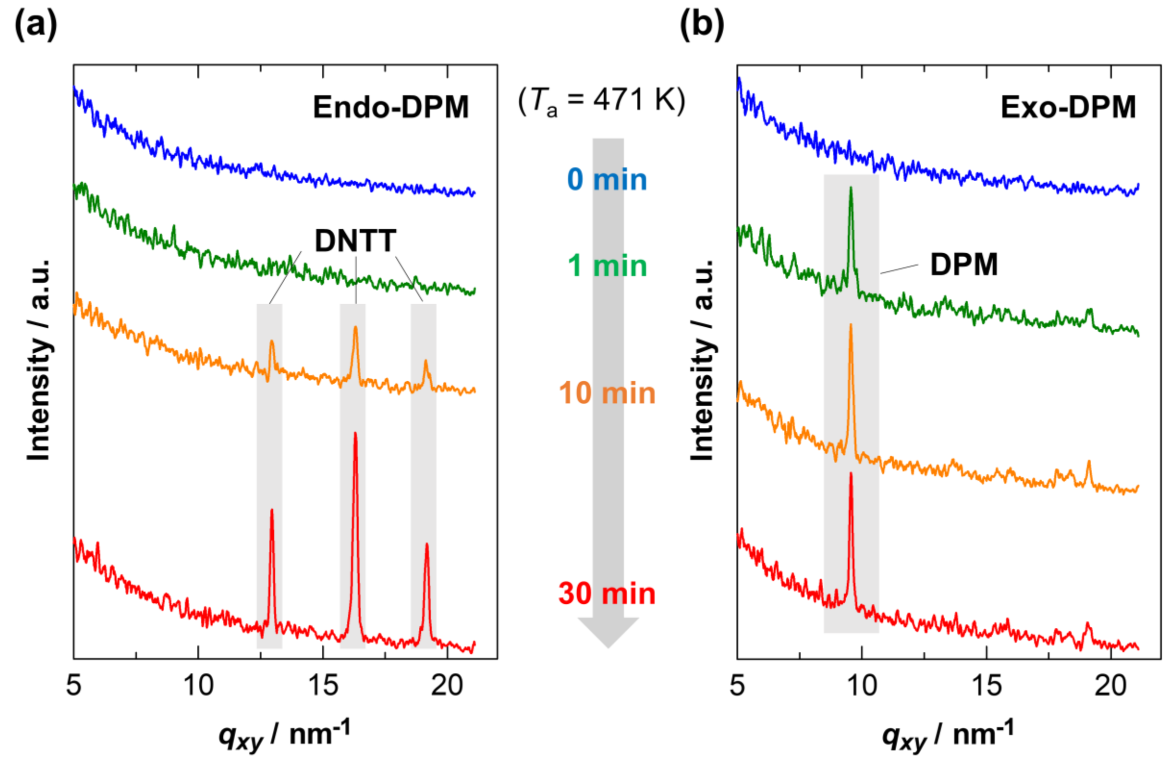

Figure S4. GIXD patterns of endo-DPM (a) and exo-DPM (b) films annealed at 471 K as a function of annealing time.

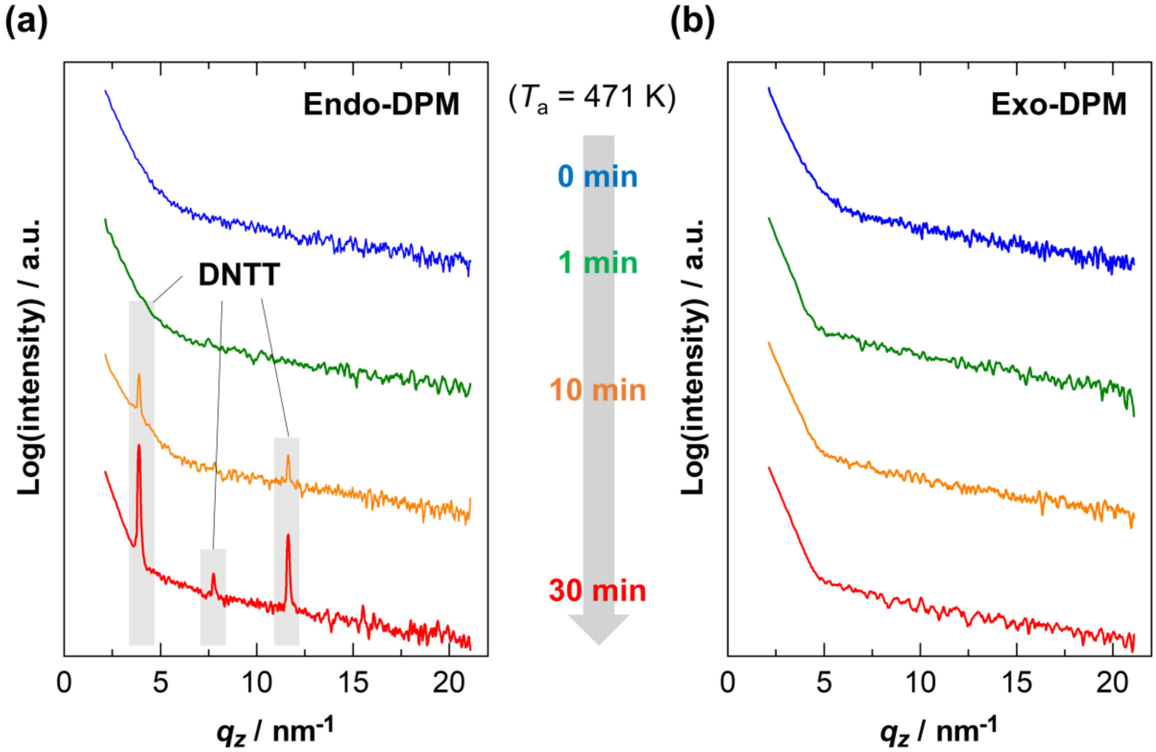

Figure S5. Specular XRD patterns of endo-DPM (a) and exo-DPM (b) films annealed at 471 K as a function of annealing time.

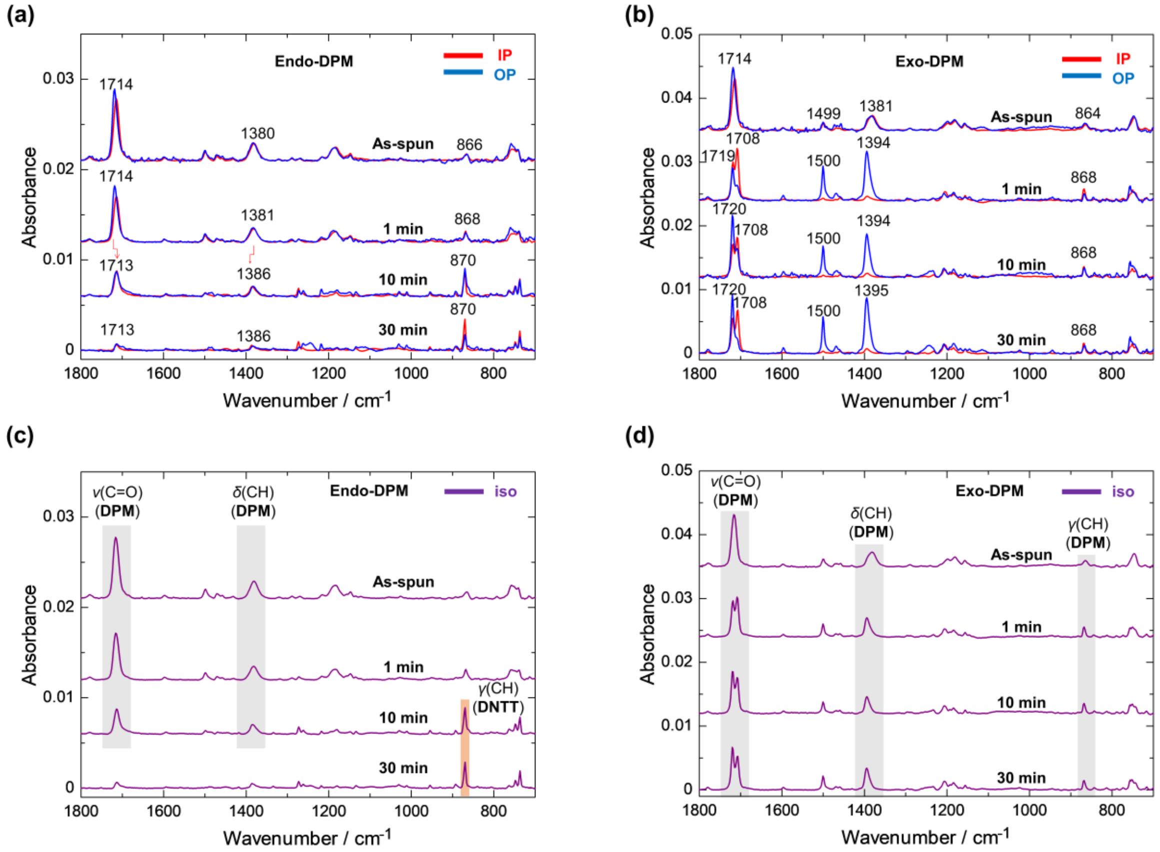

Figure S6. IR pMAIRS spectra of endo-DPM (a,c) and exo-DPM (b,d) films annealed at 471 K as a function of annealing time. The IP and OP spectra are shown in (a) and (b), and the calculated orientation-free spectra are presented in (c) and (d).
